# Unusual Cause of Knee Locking

**DOI:** 10.1155/2013/837140

**Published:** 2013-02-06

**Authors:** Gazi Huri, Omer Sunkar Biçer

**Affiliations:** ^1^Department of Orthopaedic and Traumatology Surgery, Cukurova University, Adana, Turkey; ^2^Division of Sports Medicine, Department of Orthopaedic and Traumatology Surgery, Johns Hopkins University, 10753 Falls Road, Lutherville, Baltimore, MD 21093, USA

## Abstract

We report a case of partial intrasubstance tear of popliteus tendon as an unusual cause of pseudolocking of the knee. A 13-year-old semiprofessional soccer player applied to our clinic with a locked right knee in spite of the therapy applied (cold pack, NSAID, and immobilization) in another institution 20 days after the injury. Significant extension loss was observed in his right knee with 30°–90° ROM. Magnetic resonance imaging (MRI) and arthroscopy confirmed the intrasubstance tear of popliteus tendon and synovitis. The ruptured part of the tendon was debrided, and the inflammatory tissue around the tendon, which may lead to pseudolocking, was gently removed with a shaver in order to regain the normal ROM. The patient was discharged with full ROM and weight bearing first day after the surgery. To our knowledge, this is the first case demonstrating intrasubstance tear of popliteus tendon causing pseudolocking of the knee.

## 1. Introduction

The popliteal tendon extends from the lateral femoral condyle and the fibular head into the tibia above the popliteal line. It is therefore a relatively horizontal muscle lying deep in the back part of the knee. It contributes to the stability of the knee joint by restricting posterior translation, varus rotation, and external rotation of the tibia on the femur [1]. It also functions as a dynamic internal rotator of tibia.

Popliteus ruptures are usually associated with posterolateral injuries of the knee. Although knee hemarthrosis and tenderness due to isolated rupture of popliteus tendon have been reported, locking of the knee has not been reported previously, to our knowledge. In the current case, an unusual cause of pseudolocking of the knee, which was thought to be due to muscular spasm and synovitis after popliteal tendon injury in a pediatric soccer player, is reported.

## 2. Case Report

**Figure 1:**
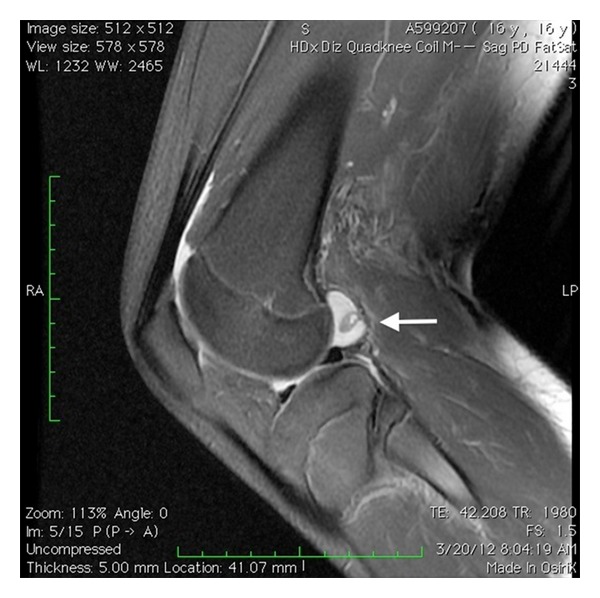
Sagittal MRI view of knee, demonstrating edema around the popliteus tendon.

**Figure 2:**
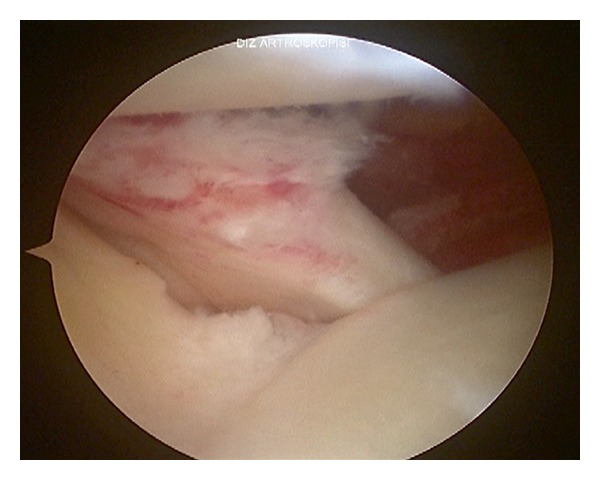
Arthroscopic view of injured popliteus tendon.

**Figure 3:**
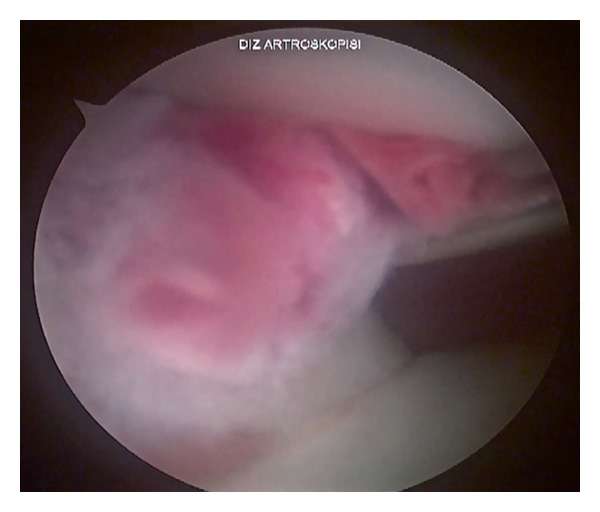
Mobilization of the intrasubstance ruptured popliteus tendon into the joint, causing impingement.

**Figure 4:**
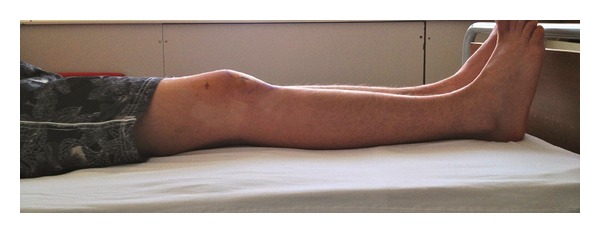
Postoperative examination of the patient with full extension.

A 13-year-old semiprofessional soccer player presented to our institution with traumatic right knee injury. He described a direct trauma to the lateral side of his partially flexed knee causing external tibial rotation. He was unable to extend his knee immediately with severe tenderness at the posterolateral side. He applied to our clinic with a locked knee in spite of the therapy applied (cold pack, NSAID, and immobilization) in another institution 20 days after the injury. Significant extension loss was observed in his right knee with 30°–90° ROM. There was mild swelling and tenderness at the lateral joint line. The ligamentous pathologies could not be evaluated completely because of severe pain during examination. Furthermore rotatory instability tests were unremarkable. MRI showed an inflammation around the popliteus tendon with no bony pathology (Figure 1). A diagnostic arthroscopic evaluation was performed in the light of these findings, 3 weeks after the injury. There was no sign of instability, and extension loss resolved in the examination under general anesthesia. These findings supported the possibility of locking caused by hamstring spasm and synovitis rather than a mechanical obstacle, though in the arthroscopic evaluation it was also detected that the popliteal tendon was partially injured causing partial impingement in the popliteal hiatus posterolaterally (Figures 2 and 3). The interfering part of the ruptured tendon was debrided, and the inflammatory tissue around the tendon was gently removed with a shaver in order to regain the normal ROM. No meniscal or ligamentous pathologies were observed. No capsular injury was detected, and the anterior cruciate ligament was found to be in continuity. The patient was discharged with a full ROM (Figure 4) and weight bearing first day after the surgery with six weeks of ongoing physical therapy.

## 3. Discussion

Several case reports of isolated popliteal tendon avulsion injuries have been published in the literature [2–5]. Regarding the previous reports, acute haemarthrosis without laxity, tenderness, and discomfort just over the popliteus tendon have been observed as main signs and findings of isolated popliteus tendon ruptures. However, popliteus tendon injuries have not been noticed as a cause of extension loss. This may be due to both an obstacle occupying the joint space as seen in true locking and hamstring contraction causing pseudolocking. To our knowledge, this is the first case that highlights the popliteus tendon injury causing locking of the knee. 

The anatomy of the popliteus tendon is well described. It arises from the proximal posterior surface of the tibia and has insertions into the posterior portion of the lateral meniscus and the femur, both deep and anterior to the lateral collateral ligament. It helps medial rotation of the knee joint and in the crouching position is the only muscle that prevents the femoral condyles from gliding forward on the tibia [6]. 

Popliteal tendon injuries are associated with knee injuries, considerably in the posterolateral corner involvement [5]; isolated injuries are fewer than 10% [7]. Its injuries are more frequently reported as femoral avulsions, and complete intrasubstance tear has been identified only in two cases [8, 9]. MRI is useful both in the diagnosis of popliteus tendon injury and additional pathologies such as posterolateral injuries. Arthroscopic assessment should be considered as a part of the diagnosis as well as the treatment [10].

Despite the small number of cases presented, there is still debate about the optimal treatment in the literature. The outcomes of the treatments including conservative treatment, open surgical procedures such as repair or reattachment with screws or anchors, and arthroscopic approaches have been clearly reported in the literature. Wheeler et al. presented cases managed conservatively with good outcomes, at least over the limited period of followup. Wheeler et al. presented cases of rupture of the popliteus tendon with an associated avulsion fracture [11]. After arthroscopic debridement and excision of bone fragment without repair of the torn tendon, complete recovery was reported at two-year followup [3]. Mirkopulos and Myer [12] reported surgical reattachment of the avulsed popliteus tendon using screw and washer. Rose and Parisien published a case that described open exploration and repair of the tendon with a good result after one year [5]. A case of isolated rupture treated with two suture anchors has been reported, with complete recovery and no signs of instability at the followup [2]. Conroy et al. reported a patient with isolated intrasubstance rupture of the popliteus tendon causing impingement, treated by arthroscopic debridement, who has returned to playing competitive soccer within 6 weeks of surgery [8].

The suspicion of popliteus tendon injury may arise from an acute haemarthrosis without laxity and pain on lateral aspect of knee or only from discomfort just over the popliteus tendon. Because of these subtle signs the pathology may easily be overlooked. Locking of the knee due to popliteus tendon injuries is not reported in the literature, to our knowledge. It may either be a true or a pseudo locking. For a knee to be truly “locked” the tendon should occupy the intercondylar space and obstruct the ACL along the roof of the notch and stop full extension. However, our case appears to be a pseudo locking due to hamstring spasm and synovitis, which has resolved under general anesthesia. 

As a conclusion, in cases with either true or pseudo locking of the knee, popliteus tendon injuries must be questioned in the differential diagnosis.
